# The Antioxidant Role of Xanthurenic Acid in the *Aedes aegypti* Midgut during Digestion of a Blood Meal

**DOI:** 10.1371/journal.pone.0038349

**Published:** 2012-06-11

**Authors:** Vitor L. A. Lima, Felipe Dias, Rodrigo D. Nunes, Luiza O. Pereira, Tiago S. R. Santos, Luciana B. Chiarini, Tadeu D. Ramos, Bernardo J. Silva-Mendes, Jonas Perales, Richard H. Valente, Pedro L. Oliveira

**Affiliations:** 1 Instituto de Química e Biotecnologia, Universidade Federal de Alagoas, Maceió, Alagoas, Brazil; 2 Instituto de Bioquímica Médica, Programa de Biologia Molecular e Biotecnologia, Universidade Federal do Rio de Janeiro, Rio de Janeiro, Rio de Janeiro, Brazil; 3 Laboratório Interdisciplinar de Pesquisas Médicas - Instituto Oswaldo Cruz, Rio de Janeiro, Rio de Janeiro, Brazil; 4 Instituto de Biofísica Carlos Chagas Filho, Universidade Federal do Rio de Janeiro, Rio de Janeiro, Rio de Janeiro, Brazil; 5 Laboratório de Toxinologia, Instituto Oswaldo Cruz, Rio de Janeiro, Rio de Janeiro, Brazil; 6 Instituto Nacional de Ciência e Tecnologia em Entomologia Molecular, Rio de Janeiro, Rio de Janeiro, Brazil; New Mexico State University, United States of America

## Abstract

In the midgut of the mosquito *Aedes aegypti,* a vector of dengue and yellow fever, an intense release of heme and iron takes place during the digestion of a blood meal. Here, we demonstrated via chromatography, light absorption and mass spectrometry that xanthurenic acid (XA), a product of the oxidative metabolism of tryptophan, is produced in the digestive apparatus after the ingestion of a blood meal and reaches milimolar levels after 24 h, the period of maximal digestive activity. XA formation does not occur in the White Eye (WE) strain, which lacks kynurenine hydroxylase and accumulates kynurenic acid. The formation of XA can be diminished by feeding the insect with 3,4-dimethoxy-N-[4-(3-nitrophenyl)thiazol-2-yl] benzenesulfonamide (Ro-61-8048), an inhibitor of XA biosynthesis. Moreover, XA inhibits the phospholipid oxidation induced by heme or iron. A major fraction of this antioxidant activity is due to the capacity of XA to bind both heme and iron, which occurs at a slightly alkaline pH (7.5-8.0), a condition found in the insect midgut. The midgut epithelial cells of the WE mosquito has a marked increase in occurrence of cell death, which is reversed to levels similar to the wild type mosquitoes by feeding the insects with blood supplemented with XA, confirming the protective role of this molecule. Collectively, these results suggest a new role for XA as a heme and iron chelator that provides protection as an antioxidant and may help these animals adapt to a blood feeding habit.

## Introduction

Feeding on vertebrate blood results in a potentially deleterious heme/iron overload in the midgut epithelium of mosquitoes [1]. Like most other hematophagous invertebrates, mosquitoes consume large amounts of blood, up to three times their own weight before the blood meal. The hydrolysis of blood proteins by midgut proteases results in the release of heme, the prosthetic group of hemoglobin. Heme is a toxic molecule because of its capacity to promote the formation of free radicals [2], [3]. When present in high concentrations, heme also induces cell lysis by a physical mechanism because, owing to its amphiphilic nature, heme can disturb the stability of phospholipid bilayers [4]. In addition, heme degradation by heme oxygenase can lead to iron release, which can promote the formation of reactive oxygen species via the Fenton reaction [5]. Both heme accumulation and heme degradation by heme oxygenase – resulting in iron release – have been shown to occur in the midgut of *Aedes aegypti* [6], [7].

Tryptophan is degraded by the kynurenine pathway, the first step of which is the oxidation of tryptophan, a reaction catalyzed by tryptophan 2,3-dioxygenase or indoleamine 2,3-dioxygenase, depending on the tissue and species studied [8], [9]. In insects, this pathway is responsible for the formation of eye pigments, the ommochromes [10]. One relatively obscure product of this pathway, xanthurenic acid (XA), has attracted much attention in recent years after being identified as the molecule that triggers *Plasmodium* differentiation inside the mosquito midgut, inducing gametocyte exflagellation via promotion of the hydrolysis of phosphatidylinositol-(4,5)-bisphosphate and the release of calcium from endoplasmic reticulum stores [11], [12]. However, despite the function performed by XA in the *Plasmodium* life cycle, its function in the physiology of the mosquito vector has not yet been elucidated.

XA has been shown to act as a peroxyl radical scavenger *in vitro*, but its function as an antioxidant *in vivo* has been considered unlikely because the concentrations that were found in the only tissue that has been studied (mouse lung) were in the low micromolar range [13]. Here, we have demonstrated the occurrence of large amounts of XA in the midgut of *Aedes aegypti* and have provided evidence for an antioxidant role of XA against an oxidative challenge based on heme or iron.

## Results

**Figure 1:**
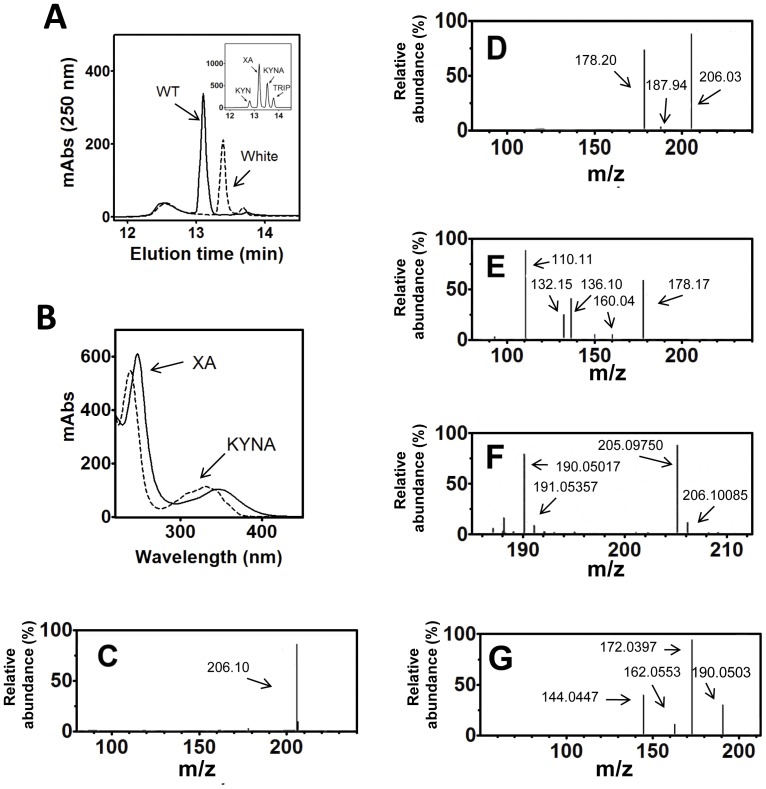
XA is an abundant component of the *Aedes aegypti* midgut. (A) HPLC profile of a midgut extract from RED strain (WT) and WE strain insects 24 h ABM (one midgut was used in each run). The inset shows an HPLC run with standards of kynurenine (KYN), xanthurenic acid (XA), kynurenic acid (KYNA) and tryptophan (TRIP). (B) Light absorption spectra of the XA peak from the WT midgut (solid line) and of the kynurenic acid peak from the WE midgut (dotted line). (C) ESI-MS of the XA [M+H]^+^ peak collected from the midgut HPLC fractionation (shown in B) with m/z 206.1 revealed a molecular mass of 205 Da. (D) MS^2^ of m/z 206.1 produced m/z 178.2 that could correspond to the loss of the formic acid plus a water addition. (E) MS^3^ of m/z 178.2 produced m/z 160.0 and 132.2 among others. (F) ESI-MS of the kynurenic acid peak collected from the WE midgut HPLC fractionation (shown in A) displaying m/z 190.050. (G) MS^2^ of m/z 190.050 produced m/z 173.000, 162.055 and 144.045, which are identical to those formed from standard kynurenic acid (not shown). The MS^3^ of the m/z 173.000 (172.0397) did not provide additional species (not shown).

**Figure 2:**
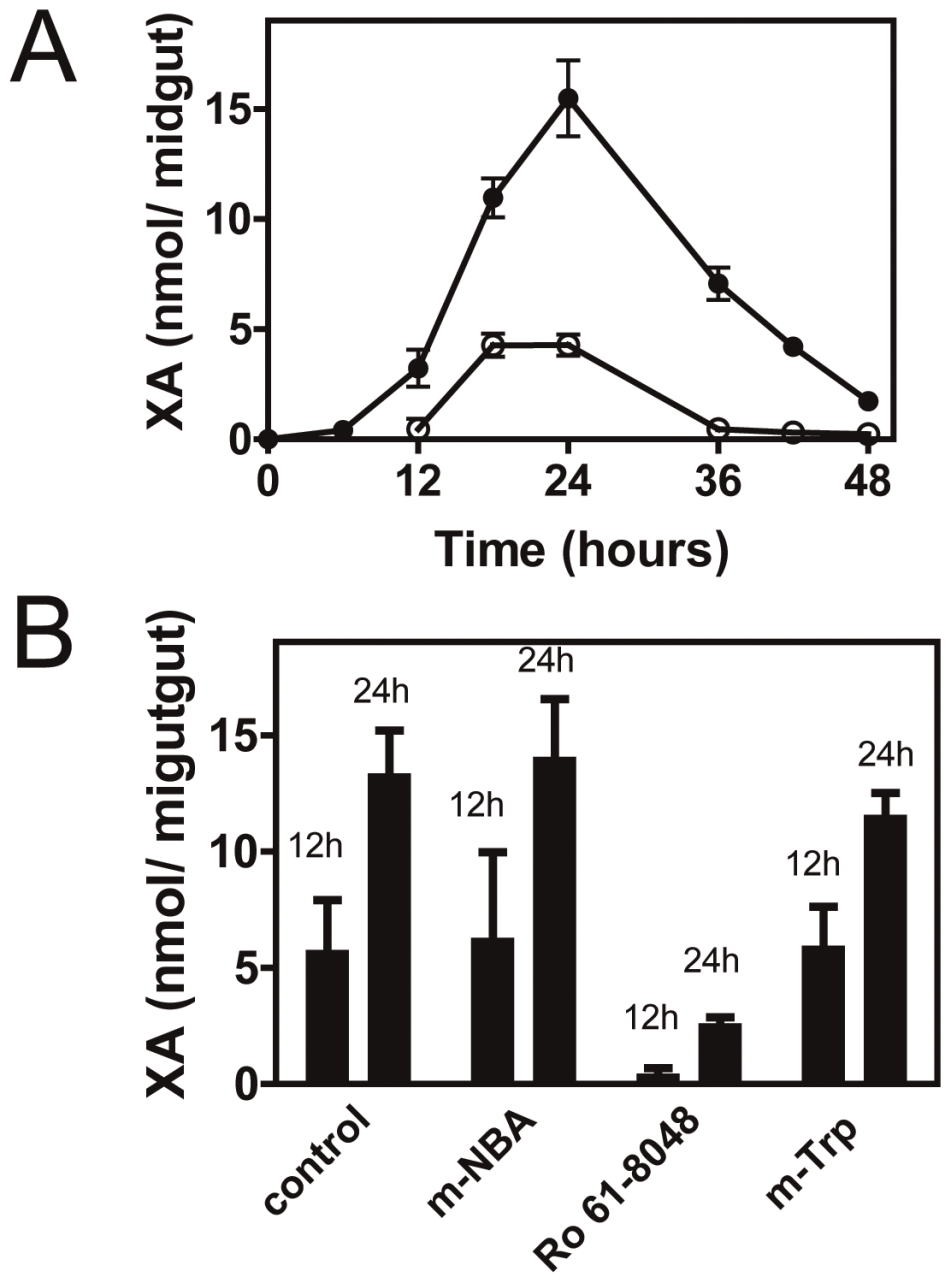
XA concentration increases in the midgut ABM owing to an increase in the kynurenine pathway. (A) The midguts of adult females were dissected after feeding the insects with rabbit blood (•) or with plasma (○) at the indicated times, and the XA content was evaluated using HPLC. (B) The insects were fed with blood plus 1 mg/ml 1-methyl-tryptophan, compound Ro-61-8048 or m-NBA, and the XA content was evaluated at 12 h and 24 h ABM. * indicates p<0.05 using Student's t-test. Data shown are mean ± SEM (n = 4).

Midgut homogenates from adult females were dissected 24 h after a blood meal (ABM) and analyzed by reverse phase HPLC. A major light absorption peak at 250 nm was identified as XA, on the basis of its retention time (Figure 1A) and the observation that its light absorption spectrum was identical to that of an XA standard (Figure 1B). Mass spectrometry analysis of this peak (Figure 1C–E) confirmed its identity as XA because the fragmentation of the [XA+H]^+^ ion (m/z 206.1) generated spectra similar to those reported by Billker *et a*l. [11], with predictable daughter ions at m/z 178.2, 160.0, 150.1 and 132.2. Homogenates of insects fed only with sugar, did not contain XA or had very low titers of XA below the detection limits of the diode array detector (<0.1 nmol). When the midguts from the WE strain were analyzed, a peak with a different mobility was found, and this species exhibited a light absorption spectrum distinct from that of XA and indistinguishable from the kynurenic acid (KA) standard spectrum (Figure 1A and B). The species giving rise to this peak was collected and shown to be authentic KA from its mass spectrum, which exhibited an ion peak at m/z 190.050 and daughter ions at m/z 172.039, 162.055 and 144.044 (Figure 1F and G). Peaks from both XA and KA from midgut showed ion peaks that were identical to those obtained upon fragmentation of standards (Spectra of standards and assignment of major ion peaks are shown in Figures S1 and S2). After a blood meal, intense formation of XA occurred, reaching a maximum at 24 h when approximately 15 nmol was accumulated in a single midgut (Figure 2A). Assuming a blood bolus volume of 3 µl [14], this accumulation of XA would result in an XA concentration of approximately 5 mM in the midgut lumen or even higher if the intense diuresis that occurs after blood ingestion is taken into account. Feeding insects with rabbit plasma alone resulted in a 70% reduction in the XA peak (Figure 2A), suggesting that the meal protein profile strongly influenced the metabolic flow in the kynurenine pathway. A reduction of XA formation by 80% was obtained by feeding insects with blood enriched with Ro-61-8048, a specific inhibitor of kynurenine 3-hydroxylase [15], confirming that the increase in XA was due to increased tryptophan degradation via the kynurenine pathway (Figure 2B). In contrast, m-NBA, which is also known to inhibit kynurenine 3-hydroxylase in mammalian cells, did not result in a significant reduction in the level of XA. A commonly used inhibitor of the tryptophan degrading enzymes indoleamine 2,3-dioxygenase and tryptophan 2,3-dioxygenase, 1-methyl-tryptophan, also did not result in a significant reduction in the XA levels in the gut. The lack of inhibition by these two compounds may reflect the specificity of the mosquito enzymes or, alternatively, may be explained by the degradation of these drugs in the midgut environment or by their low uptake by midgut cells. Choosing among these alternatives is difficult because there are no other reports on the effect of these compounds on enzymes of other insect species.

**Figure 3:**
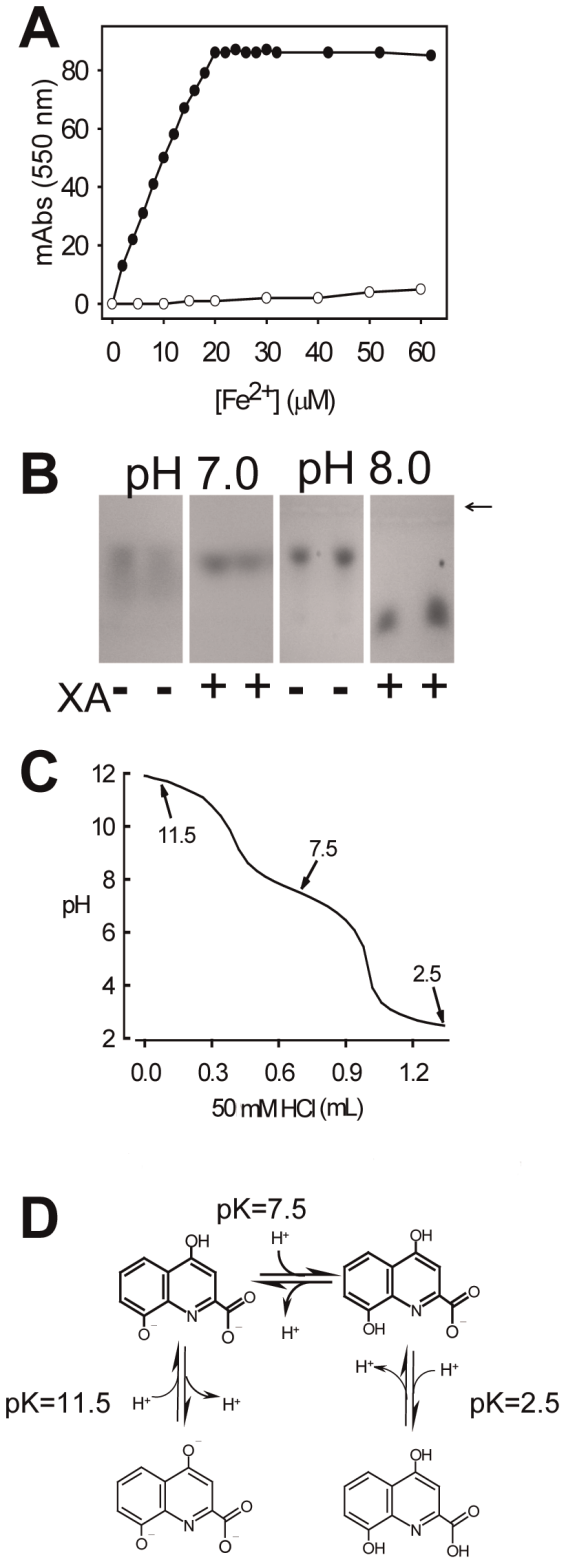
XA binds to iron and heme in a pH-dependent manner. (A) Fe_2_SO_4_ was added to XA (50 µM, filled circles), and the absorbance at 550 nm was monitored. A control in the absence of XA was evaluated (open circles). (B) 1% agarose gels were made with Na-phosphate buffer at pH 7.0 or 8.0 with or without 1.5 mM XA. The samples with 10 nmol of heme in a phosphate buffer of the same pH were applied, and the electrophoresis was performed. The arrow indicates the sample application origin. (C) Titration curve for XA. (D) Ionic forms of XA.

XA is known to be a high-affinity iron chelator [16], [17], and we show here that it is also capable of binding heme because the addition of XA to an agarose gel changes heme’s electrophoretic mobility (Figure 3). The binding of heme to XA is strongly affected by pH changes between pH 7 and 8, a pH range in which occurs the dissociation of the 8-hydroxyl group, which has a pKa of approximately 7.5 (Figure 3C and D), a result that was attributed to the binding of heme to the quinolinic ring. Due to the presence of this negatively charged group in XA at pH 8.0, its binding to the positive heme iron would result in the formation of a complex with altered eletrophoretic mobility.

**Figure 4:**
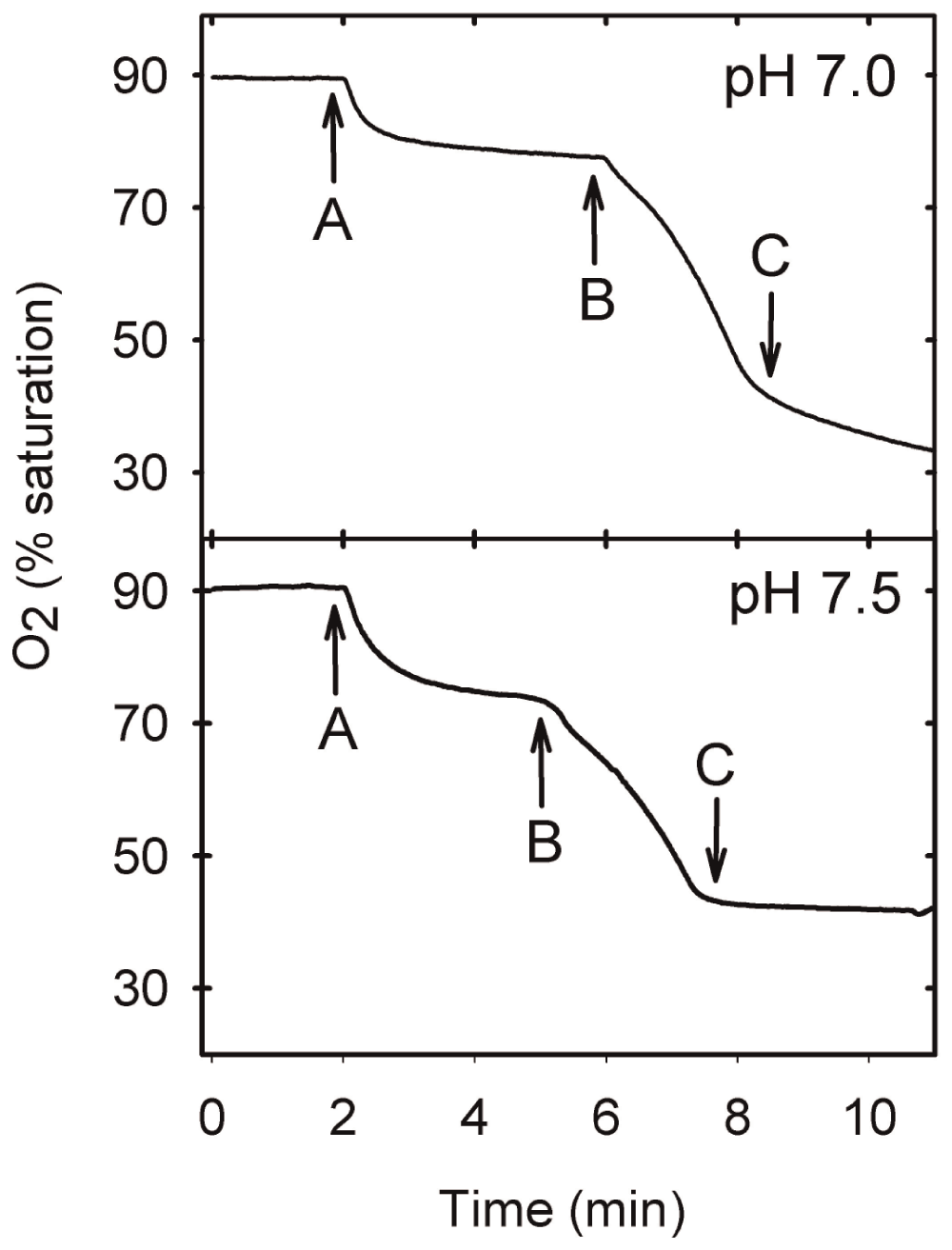
XA inhibits Fe^2+^-induced lipid peroxidation. Phospholipid micelles were prepared in TBS and 100 µM Fe_2_SO_4_, and 100 µM ascorbic acid was added (A) followed by 100 µM H_2_O_2_ (B) and then 200 µM XA (C). The experiment shown in the upper panel was performed at pH 7.0, and the experiment in the lower panel was performed at pH 7.5. The oxygen consumption by lipid oxidation was monitored with a Clark-type electrode.

**Figure 5:**
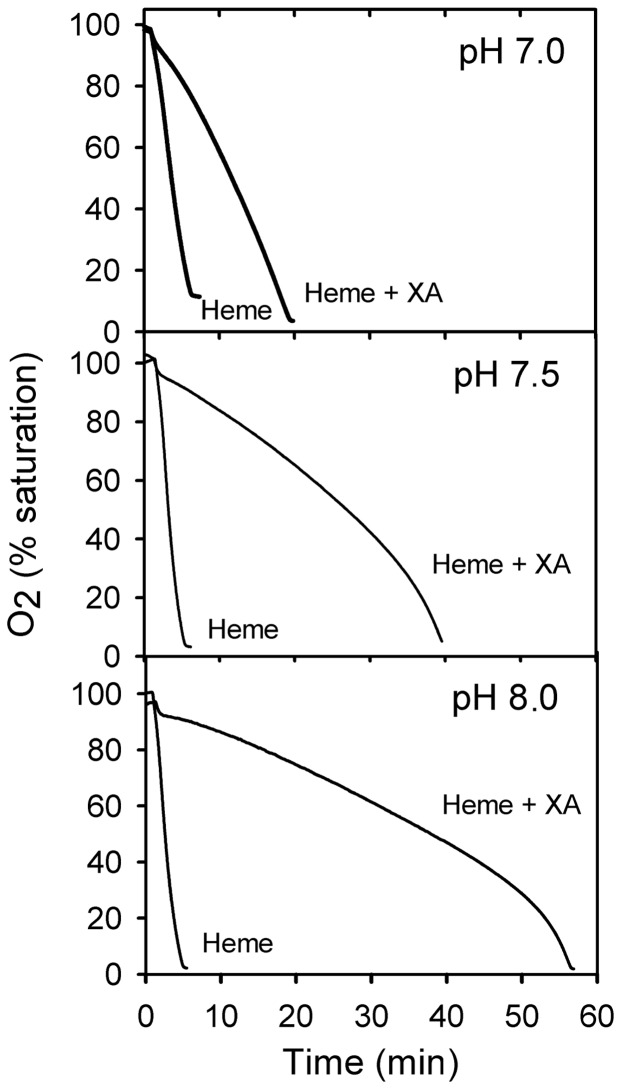
XA inhibits heme-induced lipid peroxidation. Phospholipid micelles in TBS (at pH 7.0, 7.5 or 8.0, as indicated) were supplemented with heme (3.3 µM) or heme (3.3 µM)+XA (20 µM). The oxygen consumption by lipid oxidation was monitored with a Clark-type electrode.

Iron-induced lipid peroxidation occurs via the action of the hydroxyl radicals formed by the Fenton reaction [5]. The effect of XA on the rate of oxidation of phospholipid micelles by the iron or heme–promoted Fenton reaction was evaluated following O_2_ consumption by the lipid peroxidation chain with an oxygen electrode. When iron-induced lipid peroxidation was evaluated using this approach, XA proved to be an efficient radical scavenger at pH 7.0, as shown previously for peroxyl radicals [13], but its efficiency as an antioxidant increased at pH 7.5 (Figure 4). Heme is also able to promote lipid peroxidation, but this promotion probably involves a mechanism distinct from iron-dependent lipid peroxidation [1]. Heme-induced lipid peroxidation is also inhibited by XA, and the reduction in the rate of oxygen consumption was also much more pronounced at a higher pH (Figure 5). Collectively, these experiments suggest that the action of XA as an antioxidant is improved by its capacity to bind heme and iron.

**Figure 6:**
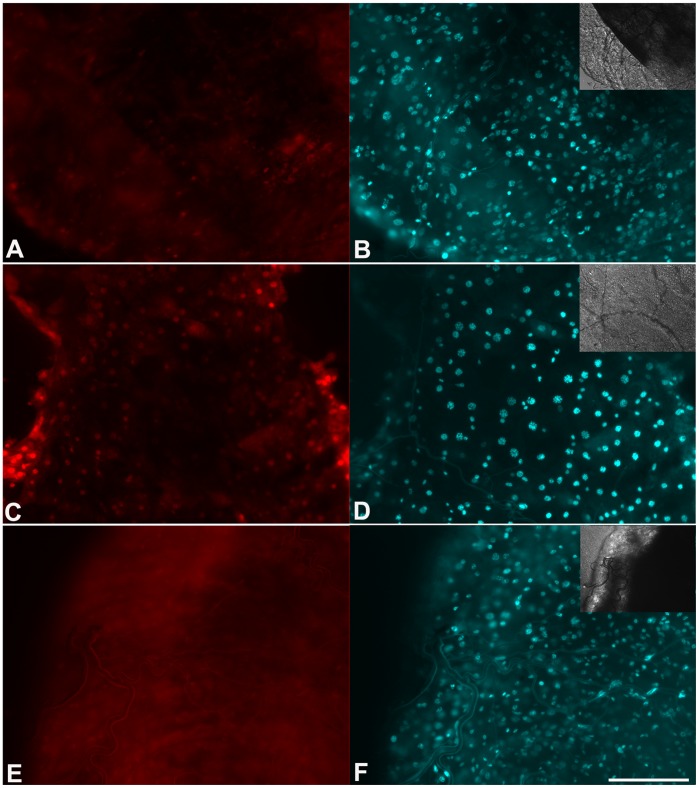
Lack of XA in the WE mosquito promotes cell death in the midgut epithelium. Midguts were dissected 24 h after blood feeding and incubated with propidium iodide to evaluate cell death. Propidium iodide and DAPI fluorescence are shown in the panels A, C, E, and B, D, F, respectively. Insets are differential interference contrast (DIC) images. A,B - RED strain; C,D - WE strain; E,F - WE strain fed on blood supplemented with XA 6 mM. Scale bar – 100 µm.

Midgut dissected from WE mutants are clearly more fragile than wild type (RED strain) mosquitoes. This observation lead us to hypothesize that increased cell death could be occurring. When tissue was incubated with propidium iodide, a fluorescent nuclear probe used to evaluate cell death by necrosis [18], much more nuclei were labeled in the WE midgut than in the epithelia from RED strain mosquitoes (Figure 6 A–D). To test if this could be explained solely by the lack of XA, insects were fed with blood supplemented with XA, which resulted in a marked reduction of cell death to levels similar to those found in wild type insects (Figure 6 E–F).

## Discussion

In insects, one of the intermediates in the kynurenine pathway, 3-hydroxykynurenine (3-HK), is directed to the formation of eye-pigments, the so-called ommochromes [19]. XA is a less-studied molecule that is frequently described as an end product of this pathway and to which no physiological function has been ascribed until now. Here, we show that XA synthesis is dramatically increased after the ingestion of a blood meal by *Aedes aegypti*, reaching maximum values at 24 h ABM, when the hemoglobin degradation by the late trypsin protease also attains its maximum rate [20]. The reduction in the XA titers that follows is explained by the excretion of XA, which is found in the feces that are excreted thereafter (data not shown).

XA has been shown to act as a scavenger of peroxyl radicals *in vitro* [13]. Here, we have evaluated the antioxidant action of XA using heme and iron as promoters of radical formation because these molecules constitute a physiological oxidative challenge during blood digestion [1]. XA proved to be a powerful antioxidant, inhibiting lipid peroxidation induced both by heme and by iron. A remarkable feature of the antioxidant activity of XA that applies equally both for iron and heme was its pH dependence, showing a marked increase inits capacity to prevent lipid peroxidation when pH changed from 7.0 to 7.5 or 8.0 (Figures 4 and 5). As this pH range coincides with the dissociation of the phenolic 8-hydroxyl group (pKa ∼ 7.5; Figure 3D), this result led us to.suggest that this pH-dependent additional antioxidant capacity involved the binding of heme and iron to XA. The hypothesis that these molecules bind to XA was confirmed by direct observation of binding of both iron (Figure 3A) and heme (Figure 3B). Interestingly, the mosquito midgut pH is approximately 7.5, reinforcing the idea that the binding of heme and iron should occur under physiological conditions in which the digestion of the blood meal actually takes place. Until now, only the binding of heme to proteins has been shown to inhibit the oxidative damage of biomolecules [1], [21]. To our knowledge, this is the first report of a physiological heme chelator of low molecular mass that works as a preventive antioxidant. Quinolobactin, a quinolinic compound derived from XA, has been assigned a physiological role as an iron chelator in some species of bacteria in which it has been shown to function as a siderophore [22]. It would be interesting to investigate whether the presence of an iron (and heme) chelator in the midgut affects the iron metabolism of both the insect [23], [24], [25] and the intestinal microbiota [26], [27], [28].

As mentioned before, low concentrations of XA (in the low micromolar range) are observed *in vivo* in vertebrate tissues, such as in mouse lung extracellular fluids and rat brain [13], [29]. Evidence for a signaling role for XA in this concentration range has been reported in rat brain [29]. Concentrations of XA in this range would allow XA to act as a promoter of *Plasmodium* exflagellation, which has a half-maximal activity at 9 µM [11], but certainly would not be enough to permit XA to function as an antioxidant [13]. Our calculated estimate for the concentration of XA found in the midgut lumen of *Aedes* is approximately three orders of magnitude (∼ 5 mM) higher, which strongly suggests a role for this compound in the digestive physiology of the mosquito. In mammalian models it has been reported that most the physiological consequences of the degradation of tryptophan could be attributed to the reduction of tryptophan level, and not to the formation of a metabolite [30]. The WE mutant, that lacks kynurenine hydroxylase, is still able to degrade tryptophan but accumulates kynurenic acid instead of XA (Figure 1). This change has a profound impact on midgut tissue physiology, as evidenced by the dramatic increase in cell death in the midgut epithelium (Figure 6). XA administration in the blood meal reduced cell death in the midgut epithelia of the mutant mosquito to levels indistinguishable from the wild type. These results clearly demonstrate that the increase in XA concentration has an important role in gut tissue homeostasis.

Because the hemoglobin polypeptide concentration in vertebrate blood is approximately 10 mM, the complete hydrolysis of hemoglobin would generate an equivalent molar concentration of heme. We reported the binding of heme to the peritrophic matrix of *Aedes* when it was estimated that the amount of heme associated with this extracellular matrix could reach 2/3 of the heme content of a 3 µl blood meal [6]. Next, we demonstrated that one of the peritrophins, which are proteins of the peritrophic matrix that were previously known to bind chitin, is also able to bind heme [31]. We have also described heme detoxification by means of heme degradation in mosquito midgut mediated by a heme oxygenase enzyme [7]. More recently, we also show that production of reactive oxygen species in the mosquito midgut is markedly down-regulated after a blood meal and we proposed that this is also an adaptation that would partially compensate for the oxidative challenge imposed by the dietary heme [32]. All these data together with the present report suggest that the adaptation of the mosquito to a high heme intake is a highly pleiotropic trait. Therefore, the binding of heme to XA appears to be a novel player among multiple protective mechanisms that complement each other to ameliorate heme toxicity.

The formation of XA has been suggested to be an adaptation to prevent the accumulation of 3-hydroxykynurenine (3-HK), one intermediate in the biosynthesis of ommochromes [19]. 3-HK has been shown to accumulate in some pathologic states [33], in which it has been associated with promoting apoptosis as a consequence of the hydrogen peroxide formation that occurs upon oxidation of 3-HK. This hypothesis, which can explain XA formation during the synthesis of eye pigments, does not apply equally well to the situation found in the midgut, where XA accumulates in the absence of ommochrome formation. The binding of heme and iron by XA, acting as an antioxidant mechanism that ameliorates the toxicity of these compounds, suggests a new role for XA. Whether this function is limited to the midgut of mosquitoes or whether XA also acts as an antioxidant in other tissues and in other insect species is a question for future research.

## Materials and Methods

### Chemicals

Hemin (Fe(III) protoporphyrin IX chloride) was purchased from Frontier Scientific (Logan, UT). XA, 1-methyl-tryptophan and phosphatidylcholine were obtained from Sigma (IL). 4-dimethoxy-N-[4-(3-nitrophenyl)thiazol-2-yl]benzenesulfonamide (Ro-61-8048) and m-nitrobenzoyl-alanine (m-NBA) were kindly donated by Dr. Stephan Röver (F. Hoffmann-La Roche, Ltd., Pharmaceuticals Division).

### Ethical Statement

All animal care and experimental protocols were conducted following the guidelines of the institutional care and use committee (Committee for Evaluation of Animal Use for Research from the Federal University of Rio de Janeiro, CAUAP-UFRJ) and the NIH Guide for the Care and Use of Laboratory Animals (ISBN 0-309-05377-3). The protocols were approved by CAUAP-UFRJ under registry #IBQM001. The technicians dedicated to the animal facility at the Institute of Medical Biochemistry (UFRJ) conducted all aspects related to rabbit husbandry under strict guidelines to ensure the careful and consistent handling of the animals.

### Mosquitoes

*Aedes aegypti* from WE strain [34] were kindly provided by Dr. Paul Howel (CDC; Atlanta, USA). However, these mosquitoes had a genetic background distinct from the RED strain, here used as the control, wild type strain. In order to have the WE mutation in the mosquitoes with same genetic background as our colony, we made 8 cycles of crossing WE x RED and recovering the white eye recessive homozygous F2 insects. *Aedes aegypti* larvae from RED or WE strains were reared on dog chow pellets (Purina). The adults were maintained on a 12 h:12 h light-dark period at 27°C and 80% relative humidity. The adults were offered a 10% sucrose solution *ad libitum*. The females were used three to five days after emergence. The insects were fed with rabbit blood, heparinized blood enriched with kynurenine pathway inhibitors or rabbit plasma. For artificial feeding, meals were offered through a Parafilm membrane stretched across the bottom of a water-jacketed glass feeder apparatus maintained at 37°C. The female midguts were dissected under 50% ethanol, homogenized in 10 mM sodium phosphate, 0.15 M NaCl (pH 7.4) (PBS) and centrifuged for 15 min at 12,000×g. The supernatants were stored at −20°C until use.

### HPLC Fractionation

HPLC was performed on a Shimadzu CLC-ODS C18 column (0.15×22 cm) using a Shimadzu LC-10AT device (Tokyo, Japan) equipped with a diode array detector (SPD-M10A). The chromatography analysis was performed using a flow rate of 1.0 ml/min. Each midgut was homogenized in a 1.5 ml polypropylene tube in 150 µl PBS, and 450 µl of buffer A (5% acetonitrile and 0.05% trifluoroacetic acid) was added. The samples were centrifuged for 15 min at 12,000×g, and a 200 µl sample was applied to the column. The chromatographic separation was performed for 5 min in buffer A followed by a 15 min linear acetonitrile gradient from 20 to 80% buffer B (80% acetonitrile and 0.05% trifluoroacetic acid) and then 15 min in 100% buffer B. The light absorption spectra were recorded during chromatography, using a diode array detector. The absorbance values at 250 nm were used to measure the levels of XA using a standard curve obtained by applying known amounts of XA to the column and performing HPLC runs under the same conditions.

### Mass Spectrometry

The mass spectra of the samples obtained from wild type Red strain mosquitoes and the XA standards were acquired in the positive-ion mode using a Finnigan LCQ Deca XP Plus ion trap mass spectrometer (ThermoElectron Co., San Jose, CA). The HPLC midgut fraction, corresponding to the commercial XA retention time, was prepared in 50% acetonitrile and 0.1% formic acid and injected by direct infusion. The spray source and capillary voltages were set to 4.5 kV and 40 V, respectively; the capillary temperature was 200°C. The collision-induced fragmentation (tandem ESI-MS) of the parent ions was performed using a relative collision energy of 40%. The mass spectra for the samples obtained from WE Aedes aegypti and standard kynurenic acid were acquired in the positive-ion mode using a Finnigan LTQ Orbitrap XL mass spectrometer (ThermoElectron Co., San Jose, CA). The HPLC midgut fraction, corresponding to the commercial kynurenic acid retention time, was prepared in 50% acetonitrile and 0.1% formic acid and injected by direct infusion. The spray source and capillary voltages were set to 4 kV and 26 V, respectively; the capillary temperature was 275°C. The collision-induced fragmentation (tandem ESI-MS) of the parent ions was performed using a relative collision energy of 40%. All spectra (MS and MS/MS) were acquired in the Orbitrap analyzer using a resolution setting of 100,000.

### Oxygen Consumption

The liposomes of soybean phosphatidylcholine (12 mg/ml were prepared in 1.5–3.0 ml of 0.15 M NaCl, 10 mM Tris-HCl buffer (TBS) at pH 7.0, 7.5 or 8.0 via 6 sonication cycles of 20 s at 4°C using a Branson Sonifier mod. 250 (Danbury, CT) set at 80 W. The oxygen consumption was measured using a Clark-type electrode (YSI, model 5300, Yellow Springs, OH) calibrated to 100% with air-saturated buffer at 25°C. Fresh heme stock solutions (10 mM) were prepared in 0.1 M NaOH, diluted to 100 µM in TBS at the indicated pH (pH 7–8) and diluted to 3.3 µM in 1.5 ml of the same buffer in the electrode cell chamber.

### Titration of XA

Potentiometric titration of 5 ml of 7 mM XA solution was performed by bringing the solution to pH 11.9 by adding 40 µl of 1N NaOH, and then progressively adding of 0.05 N HCl while recording pH with a pHmeter.

### Heme Binding to XA

The interaction of heme with XA was monitored in 1.0% agarose gels made in 50 mM sodium phosphate buffer, pH 7.0 or 8.0, in the presence or absence of 1.5 mM XA. The heme samples were applied to the gels (10 µl of a 1 mM solution of heme in 10% PEG 3350 and 50 mM Na- phosphate buffer of the same pH used in the gel), and the gels were run for 120 min at 55 V using a horizontal electrophoresis apparatus. As heme is a colored molecule, its visualization did not need staining and bands were photographed immediately at the end of the run.

### Iron Binding to XA

The association of iron with XA was evaluated by the formation of a green complex [35] by the sequential addition of a freshly prepared 1 mM Fe_2_SO_4_ solution to 1 ml of a solution of 1.5 mM XA in 10 mM Tris-HCl, pH 7.5, while following the formation of the green iron-XA complex via the increase in the absorbance at 550 nm using a GBC 920 UV-VIS spectrophotometer (Victoria, Australia).

### Cell Death Evaluation

RED or WE strains were artificially fed on rabbit blood, supplemented or not with XA 6 mM. Midguts were dissected and transferred to a 24-well tissue culture flask containing 100 µl of Schneider's medium with propidium iodide 7.5 µM and incubated for 1 h at dim light/room temperature in a high humidity chamber. Uptake of propidium iodide by cells indicates loss of membrane integrity [18]. Midguts were transferred to a drop of Vectashield Mounting Medium (Vector, Southfield, MI) with DAPI (4-6-di-amino-2-phenylindole). on a glass slide and examined with a Zeiss AxioObserver with an Axiocam MRC5 using a Zeiss-15 filter set (excitation BP 546/12; beam splitter FT 580; emission LP 590, for propidium iodide) or Zeiss-01 filter (excitation BP 365/12; beam splitter FT 395; emission LP 397, for DAPI). Differential interference contrast (DIC) images were acquired with the same microscopy.

## Supporting Information

Figure S1 **Spectra of XA standard acquired in the positive-ion mode using a Finnigan LCQ Deca XP Plus ion trap mass spectrometer.** XA was prepared in 50% acetonitrile and 0.1% formic acid and injected by direct infusion. (A) MS^2^ of m/z 206.1 produced m/z 178.2 that is explained by loss of the formic acid plus a water addition and m/z 187.9 that correspond to loss of one hydroxyl. (B) MS^3^ of m/z 178.2 produced m/z 160.0 (loss of formic acid) and 132.2 (loss of the formic acid, one hydroxyl and the nitrogen plus one H^+^). Numbers in red are m/z values expected for the fragment indicated, numbers in black are m/z obtained in the spectrometer.
(TIF)

Figure S2 **Mass spectra (MS^2^ of 190.0505) of kynurenic acid standard was acquired in the positive-ion mode using a Finnigan LTQ Orbitrap XL mass spectrometer.** Kynurenic acid was prepared in 50% acetonitrile and 0.1% formic acid and injected by direct infusion. Numbers in red are m/z values expected for the species indicated, numbers in black are experimental m/z obtained in the spectrometer.
(TIF)
