# Supplementary figures and images for: The Antioxidant Role of Xanthurenic Acid in the Aedes aegypti Midgut during Digestion of a Blood Meal
Source: PLoS One. 2012 Jun 11;7(6):e38349. doi: 10.1371/journal.pone.0038349 (PMC3372515; doi:10.1371/journal.pone.0038349)

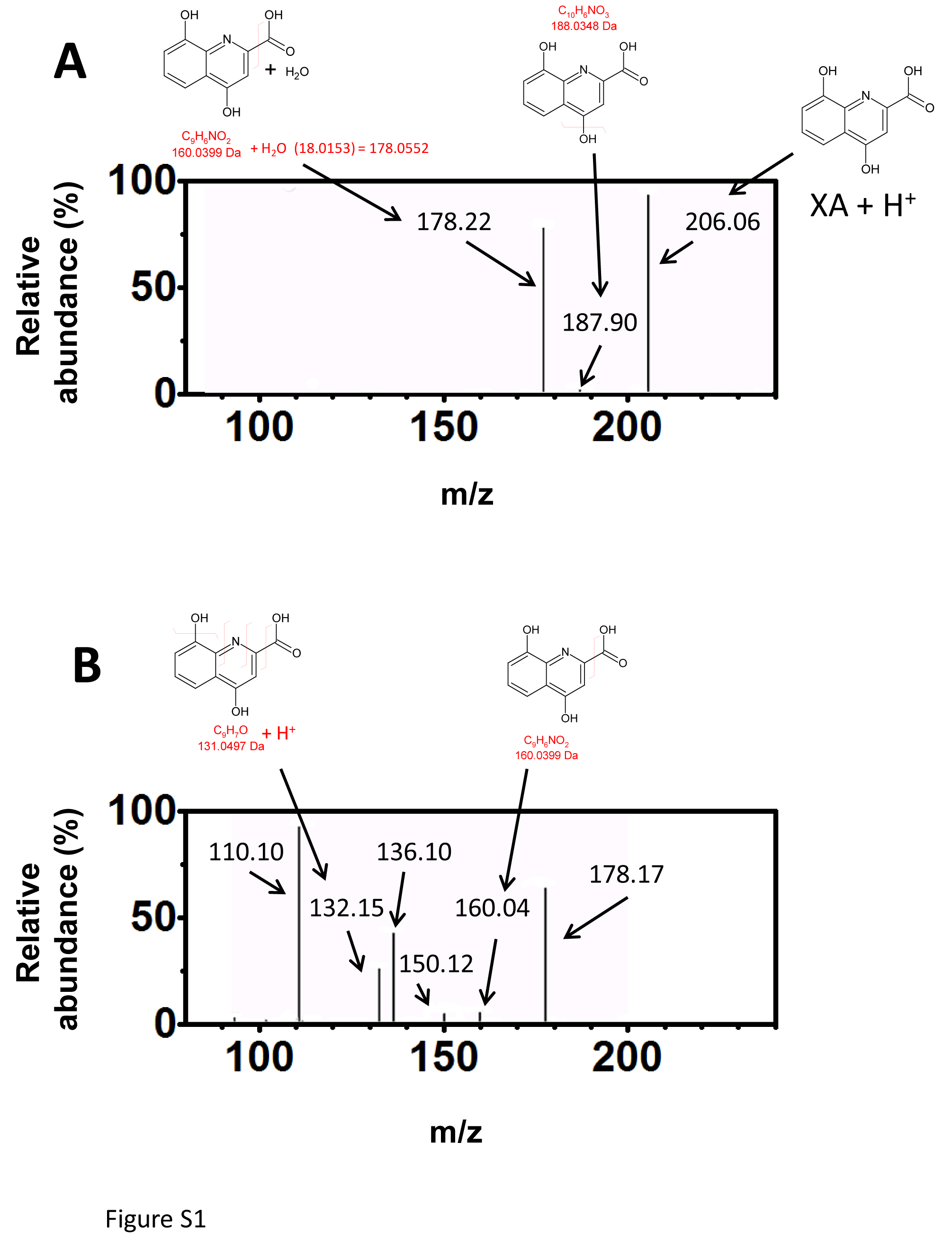

Supplement: Figure S1 — Spectra of XA standard acquired in the positive-ion mode using a Finnigan LCQ Deca XP Plus ion trap mass spectrometer. XA was prepared in 50% acetonitrile and 0.1% formic acid and injected by direct infusion. (A) MS2 of m/z 206.1 produced m/z 178.2 that is explained by loss of the formic acid plus a water addition and m/z 187.9 that correspond to loss of one hydroxyl. (B) MS3 of m/z 178.2 produced m/z 160.0 (loss of formic acid) and 132.2 (loss of the formic acid, one hydroxyl and the nitrogen plus one H+). Numbers in red are m/z values expected for the fragment indicated, numbers in black are m/z obtained in the spectrometer. (TIF) [file pone.0038349.s001.tif]

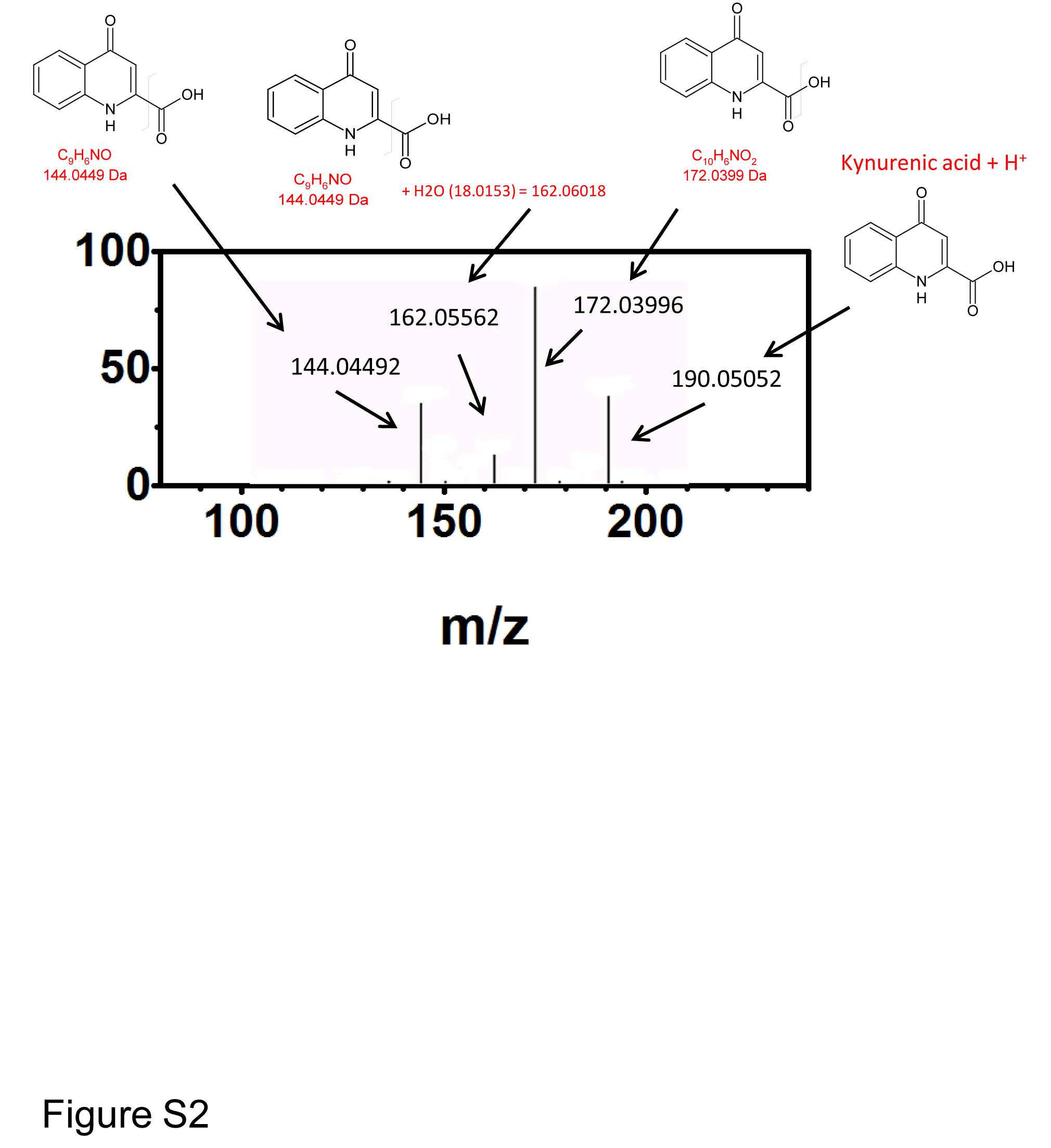

Supplement: Figure S2 — Mass spectra (MS2 of 190.0505) of kynurenic acid standard was acquired in the positive-ion mode using a Finnigan LTQ Orbitrap XL mass spectrometer. Kynurenic acid was prepared in 50% acetonitrile and 0.1% formic acid and injected by direct infusion. Numbers in red are m/z values expected for the species indicated, numbers in black are experimental m/z obtained in the spectrometer. (TIF) [file pone.0038349.s002.tif]
